# Novel Quantitative Analysis Using Optical Imaging (VELscope) and Spectroscopy (Raman) Techniques for Oral Cancer Detection

**DOI:** 10.3390/cancers12113364

**Published:** 2020-11-13

**Authors:** Ming-Jer Jeng, Mukta Sharma, Lokesh Sharma, Shiang-Fu Huang, Liann-Be Chang, Shih-Lin Wu, Lee Chow

**Affiliations:** 1 Department of Electronic Engineering, Chang Gung University, Taoyuan 333, Taiwan; mjjeng@mail.cgu.edu.tw (M.-J.J.); mukta.shrm@gmail.com (M.S.); 2 Department of Otolaryngology-Head and Neck Surgery, Chang Gung Memorial Hospital, Linkou 244, Taiwan; 3 Department of Computer Science and Information Engineering, Chang Gung University, Taoyuan 333, Taiwan; engglucky@gmail.com (L.S.); slwu@mail.cgu.edu.tw (S.-L.W.); 4 Department of Public Health, Chang Gung University, Taoyuan 333, Taiwan; 5 Graduate Institute of Clinical Medical Sciences, Chang Gung University, Taoyuan 333, Taiwan; 6 Green Technology Research Center, Chang Gung University, Guishan, Taoyuan 333, Taiwan; 7 Department of Cardiology, Chang Gung Memorial Hospital, Taoyuan 333, Taiwan; 8 Department of Physics, University of Central Florida, Orlando, FL 32816, USA; Lee.Chow@ucf.edu

**Keywords:** autofluorescence, cryopreserved tissue, oral cancer, Raman spectroscopy, PCA–LDA and PCA–QDA

## Abstract

**Simple Summary:**

Our study aims to develop a novel quantitative analysis method that can increase the oral cancer detection rate for screening oral cancer. We used two different optical techniques, a light-based detection technique (VELScope) and a vibrational spectroscopic technique (Raman spectroscopy). First, we analyzed and evaluated the performance of these two techniques individually using PCA–LDA, and PCA–QDA classifiers. The PCA–LDA of Raman spectroscopy had 82.9% accuracy, 80% sensitivity, and 85.7% specificity, while the region of interests on the autofluorescence images were differentiated with 90% accuracy, 100% sensitivity, and 80% specificity. Afterward, we combined both techniques and evaluated their performance. The combination of two optical techniques can differentiate the cancer and normal groups with 97.14% accuracy, 100% sensitivity, and 94.3% specificity. The main advantage of our study is that we can confirm our results by using two different techniques that are completely independent of each other. That is the reason that the combination of two techniques can increase the sensitivity and specificity.

**Abstract:**

In this study, we developed a novel quantitative analysis method to enhance the detection capability for oral cancer screening. We combined two different optical techniques, a light-based detection technique (visually enhanced lesion scope) and a vibrational spectroscopic technique (Raman spectroscopy). **Materials and methods:** Thirty-five oral cancer patients who went through surgery were enrolled. Thirty-five cancer lesions and thirty-five control samples with normal oral mucosa (adjacent to the cancer lesion) were analyzed. Thirty-five autofluorescence images and 70 Raman spectra were taken from 35 cancer and 35 control group cryopreserved samples. The normalized intensity and heterogeneity of the 70 regions of interest (ROIs) were calculated along with 70 averaged Raman spectra. Linear discriminant analysis (LDA) and quadratic discriminant analysis (QDA) were used with principal component analysis (PCA) to differentiate the cancer and control groups (normal). The classifications rates were validated using two different validation methods, leave-one-out cross-validation (LOOCV) and *k*-fold cross-validation. **Results:** The cryopreserved normal and tumor tissues were differentiated using the PCA–LDA and PCA–QDA models. The PCA–LDA of Raman spectroscopy (RS) had 82.9% accuracy, 80% sensitivity, and 85.7% specificity, while ROIs on the autofluorescence images were differentiated with 90% accuracy, 100% sensitivity, and 80% specificity. The combination of two optical techniques differentiated cancer and normal group with 97.14% accuracy, 100% sensitivity, and 94.3% specificity. **Conclusion:** In this study, we combined the data of two different optical techniques. Furthermore, PCA–LDA and PCA–QDA quantitative analysis models were used to differentiate tumor and normal groups, creating a complementary pathway for efficient tumor diagnosis. The error rates of RS and VELcope analysis were 17.10% and 10%, respectively, which was reduced to 3% when the two optical techniques were combined.

## 1. Introduction

Oral cancer is one of the most common cancers worldwide and is closely associated with smoking, drinking alcohol, chewing tobacco, and consuming betel quid [1]. It accounts for 90% of the oral malignancies among the 300,000 cases diagnosed annually [2]. According to Stewart [3], approximately 60% of new cases of oral cancer and 68% of deaths related to oral cancer occur in Asia. Taiwan has one of the world’s highest incidence rates of oral cancers [4]. The most common oral cancer is squamous cell carcinoma (SCC), which is usually diagnosed late, resulting in an overall five-year survival rate of 50% [5]. Early diagnosis and timely treatment may prevent oral potentially malignant disorders (OPMDs) from transforming into oral cancer [6]. Although biopsies are the gold standard for diagnosing oral cancer, they require an incision and are therefore invasive and painful. Biopsies are also time-consuming; therefore, clinicians increasingly favor non-invasive techniques such as light-based detection methods and other optical diagnostic technologies [7]. Optical imaging techniques have become adjunctive tools in oral cancer screening and utilize tissue autofluorescence arising from endogenous chromatophores to identify the presence of malignant tissue. The visualization of normal and abnormal tissues through use of optical imaging methods can improve the visual perception. These optical methods can exploit differences in the optical properties of tissues, such as fluorescence, reflectance, and chemiluminescence. The main fluorophores in the range of 400–460 nm are nicotinamide adenine dinucleotide (NADH), flavin adenine dinucleotide (FAD), cellular coenzymes, collagen, and elastin in connective tissue. The Visually Enhanced Lesion Scope (VELscope) is a handheld device that increases the visibility of oral membrane abnormalities by activating tissue fluorescence. Evidence of the capability of light-based detection methods for oral cancer screening is contradictory [8]. Previously, we reported a quantitative analysis method to classify autofluorescence images using the VELscope, thus improving use of the VELscope [9]. This quantitative analysis method can successfully differentiate normal, premalignant, and malignant lesions.

Raman spectroscopy (RS) is a vibrational spectroscopic technique. It is one of the most widely used non-invasive techniques for the non-destructive characterization of molecules and other material [10]. RS investigates the vibrational modes of a molecule that are sensitive to its chemical bonds and provides a unique “fingerprint” that enables the identification of chemicals [11]. RS has become a common technique in biological and medical applications in the early diagnosis of various types of cancer [12,13,14]. Some studies have explored the efficacy of RS to distinguish between normal and abnormal and between normal, premalignant, and malignant forms of oral mucosa using various preservation techniques and analytical methods [15,16,17,18,19,20,21]. We previously investigated the efficacy of RS (with an excitation wavelength of 532 nm) for subsite (tongue, buccal mucosa, and gingiva) oral cancer detection [22]. In that study, we used cryopreserved fresh tissue sample to classify normal and tumor tissues. Light-based detection and optical diagnostic techniques have great potential for screening and monitoring OPMDs [7]. Currently, there is no standalone method that can accurately identify OPMDs.

In this study, we combined two optical techniques to detect oral cancer; we obtained autofluorescence images using a VELscope (400–460 nm wavelength) and a vibrational spectroscopic technique using RS that employs a 532 nm laser source of excitation. Based on the autofluorescence images, we determined the intensity and the heterogeneity of lesions, while the RS detected biochemical perturbations in tissue based on the scattering of light by the vibrating molecules. Heterogeneity is calculated by the standard deviation of the lesions; it is an important feature in analyzing images, as it helps one distinguish between normal and abnormal lesions. The alteration of cellular metabolism in cancer patients is a prominent marker of tumor heterogeneity [23]. Oral cancer tissues have higher heterogeneity compared to normal or healthy mucosa. In Raman spectra, the intensity of the measured Raman scattering versus the Raman shift (wave numbers) is plotted. To achieve chemical information, the chemical bond vibration frequencies are measured in wave numbers. We have computed intensities and the heterogeneity of the region of interest (ROI) from autofluorescence images. These two different optical sources (light-based detection and vibrational spectroscopic technique) have been used to excite the fluorophores, such as NADH and FAD, and to exploit biochemical characteristics in tissues/organs. Our novel approach (combining the two optical techniques) can enhance the differentiation efficacy between abnormal and normal lesions with the highest sensitivity and specificity. This work is the first attempt to enhance the accuracy of a system by combining two different optical techniques.

## 2. Materials and Methods

### 2.1. Patients and Data Collection

**Figure 1:**
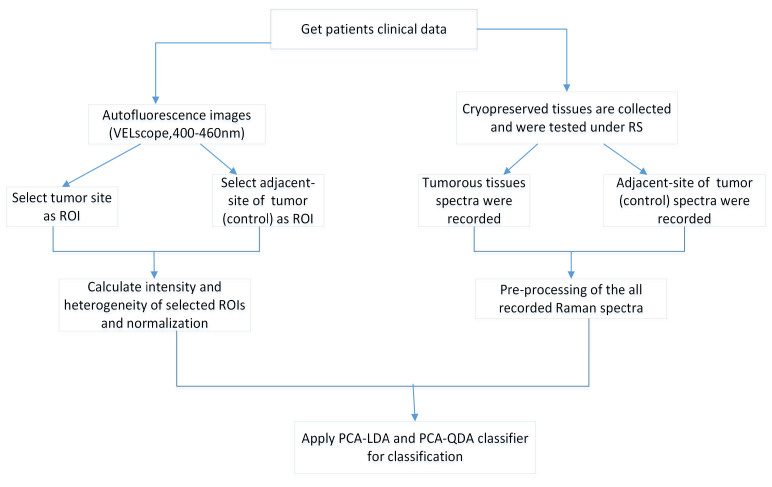
Flowchart of the proposed method.

**Figure 2:**
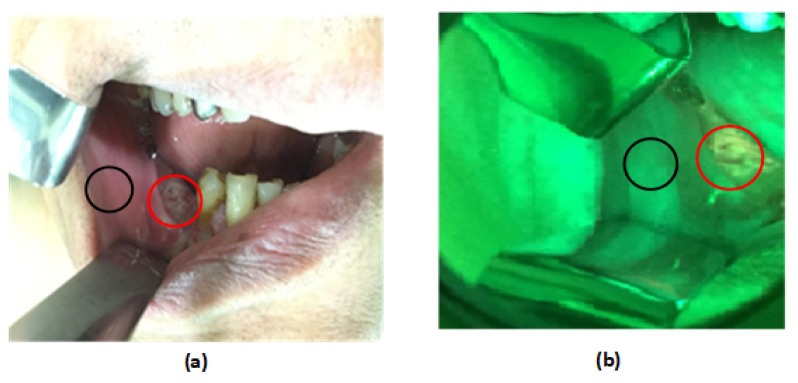
Lower gum cancer region of interest (ROI) denoted by a red circle and adjacent site of tumor (normal region) denoted by a black circle (**a**) under white light and (**b**) under the VELscope.

**Figure 3:**
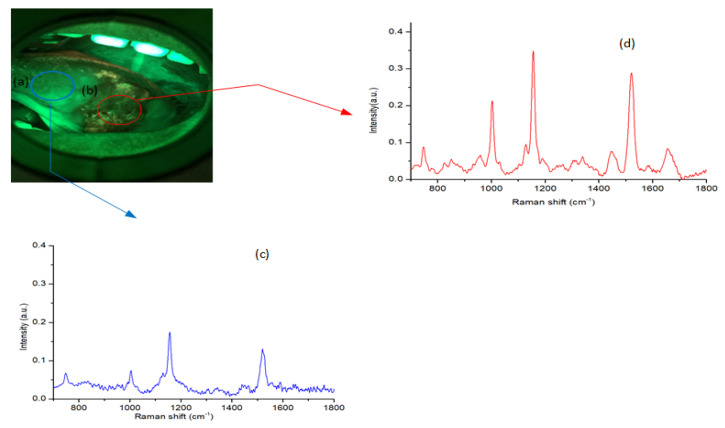
Autofluorescence image with selected ROI: (**a**) normal, (**b**) tumor. The mean of recorded Raman spectra for cryopreserved tissues: (**c**) normal, (**d**) tumor.

This study was approved by the Institutional Review Board (IRB) of the Chang Gung Medical Foundation (IRB No: 201800420B0 and 201801960B0) in Taiwan. It was performed in the Department of Otolaryngology—Head and Neck Surgery with the written and informed consent of the enrolled participants. All specimens, autofluorescence images, and pathology reports were collected at Chang Gung Memorial Hospital for analysis. Thirty-five oral cancer patients who had histologically proven malignancies were tested. One normal sample and one cryopreserved tumor tissue sample were collected from each patient. Table 1 summarizes the demographics of the thirty-five patients who were classified by their lesions at different subsites. Normal tissues (control group) were taken from a site adjacent to the tumor at the time of surgery. The study period was from Feb 2017 to July 2019. Figure 1 shows the block diagram of our proposed method of analysis. First, we collected all patient data from the hospital. All cryopreserved specimens were collected for the RS analysis. Seventy samples were collected from different subsites such as the tongue, buccal mucosa, gingiva, and mouth floor. All the specimen were at least 3×3 mm in size. The tumor samples were obtained immediately after surgery, while surgically resected tissues adjacent to the tumor (normal-appearing mucosa) were obtained 15–30 mins following surgical excision. The distance from the tumor border to the adjacent tissue was 1.5 to 2 cm. A clinician checked this margin by frozen section to ensure there was no tumor or premalignancy in the resection periphery. The cryopreserved samples were kept in liquid nitrogen (N${}_{2}$) at −80 °C immediately after the surgery to prevent changes in morphology. This method does not affect the measurements [24]. Each sample was analyzed by RS. Five spectra of each tissue sample were recorded at different locations due to the heterogeneous nature of the tissue. A total of 350 spectra were recorded from tumor and normal tissues (each per 175), yielding 70 averaged spectra (70 = 350/5). The same patients of malignancy were captured under the VELscope, and 35 autofluorescence images were collected for analysis. Thirty-five autofluorescence images with 70 ROI images (35 of tumor and 35 from adjacent site to the tumor or control group) were recorded. Figure 2 shows a autofluorescence image with two selected ROIs (tumor and control). All the ROIs from autofluorescence images were selected by the clinician (Dr. S.F. Huang). Figure 3 shows an example of a recorded autofluorescence image with selected ROIs (normal and tumor) and measured Raman spectra.

### 2.2. Preprocessing and Data Analysis Methods

Data processing and analysis were performed using MATLAB (R2018a, MathWorks, MA, USA). For Raman data, a Savitsky–Golay filter (of order 3) was used to smooth the recorded spectra to remove interference. Then, baseline correction was performed, and normalized spectra were used to eliminate data redundancy. Similarly, for autofluorescence images (VELscope data), the intensity and standard deviation for each ROI were calculated. Then, normalized value of intensity and standard deviation was enumerated to neutralize the autofluorescence effect in the ROI that arose from the parts of the image outside the ROI. These parts could include teeth, the supporting device, a prosthesis or prostheses, or filling materials.

### 2.3. Raman Spectroscopy (RS)

Each sample was placed on a glass substrate, and spectra were recorded using an RS instrument (ProTrusTech co. LTD, Taiwan). This system consisted of a laser (532 nm wavelength) as an excitation source with a maximum power of 126 mW. The spectral acquisition parameters were: laser power: 6.3 mW∼12.6 mW; integration time: 5 seconds; acquisition time: 15s. The average value of the spectrum was 3 (averaged over three spectra). The spectra resolution specified by the manufacturer was 1 cm${}^{-1}$. The laser spot size was 6 ∼ 8 micron. Five spectra were acquired from each sample for a total of 350 spectra from 70 samples. Seventy cryopreserved samples of cancer and control (normal) groups were tested randomly.

### 2.4. VELscope

The autofluorescence images were taken using the VELscope Vx (LED Dental and Apteryx, Atlanta Georgia, GA, USA). To capture the images, the VELscope was adapted with an eight-million-pixel iPod Touch (Apple, Atlanta, GA, USA).

### 2.5. Analysis Method

Principal component analysis (PCA) was used to reduce the number of dimensions. It provides principal components (coordinates) based on new dimensions. The number of PCA components was less than the half of minimal sample classes to avoid overfitting [25]. Linear discriminant analysis (LDA) and quadratic discriminant analysis (QDA) classifiers were used to study the boundary between classes and probabilities of classification; these techniques enhance the separation boundary of between class variance and within class variance. Due to this, the data variation increases in the same class and detachment between classes. The LDA classifier has a common covariance matrix with linear generated boundary, while the QDA classifier has a separate covariance matrix for each class with a quadratic boundary. QDA optimally distinguishes between the classes in the data set [26] and requires a huge data set. Therefore, LDA is more suitable for equal class samples to compare imbalanced data sets, and QDA is more suitable for unequal class samples [27]. However, in some cases they perform worse than expected [28]. To evaluate the performance of classifiers, the classifier results were validated using different validation methods.

Unsupervised PCA was applied on the normalized spectrum from 700 to 1800 cm${}^{-1}$ and to the VELscope data. The first three principal components (PC1, PC2, and PC3) generated by normalized spectrum accounted for up to 97% variance, as evaluated by PCA. The first two principal components (PC1, PC2) were fed to multivariate supervised classifier models LDA and QDA. For PCA–LDA and PCA–QDA, scores of factor 1 and 2 were chosen to obtain a scatter plot with a decision boundary. The analysis broadly categorized normal and tumor tissues. Due to the heterogeneity of the tissue, the measured spectra at the various points varied greatly in intensity and Raman shift.

We analyzed the data generated by the autofluorescence images in the same manner. Two principal components (PC1, PC2) generated by the VELscope data accounted for up to 100% variance, as evaluated by PCA. First, we analysed and evaluated the data type generated by VELsope and RS individually using PCA–LDA and PCA–QDA classifiers. Second, we combined both data types and evaluated them by selecting four principal components (PC1 and PC2 from Raman data and PC1 and PC2 from VELscope images). Afterwards, these PCs were feed in to LDA and QDA classifiers.

In our analysis, LDA and QDA shows approximately the same results. This depends on the data set and number of samples used. Other studies have had the same results in performing LDA and QDA [29]. The classifier models LDA and QDA used to divide between classes and their probabilities, while enlarging between-class variance and within-class variance. The LDA and QDA assumes a common and individual co-variance matrix of each class. This results in LDA and QDA being good classifiers for equal and unequal class samples, respectively [30]. In this study, we have an equal number of class samples for cancer and normal tissues.

## 3. Results and Discussion

A total of 70 Raman spectra and 70 ROI images (35 tumor and 35 normal) from autofluorescence images were analyzed. The spectral features with vibrational molecules and tissue fluorescence behaviour of the selected subsites are described below.

### 3.1. RS Band Spectral Features

**Figure 4:**
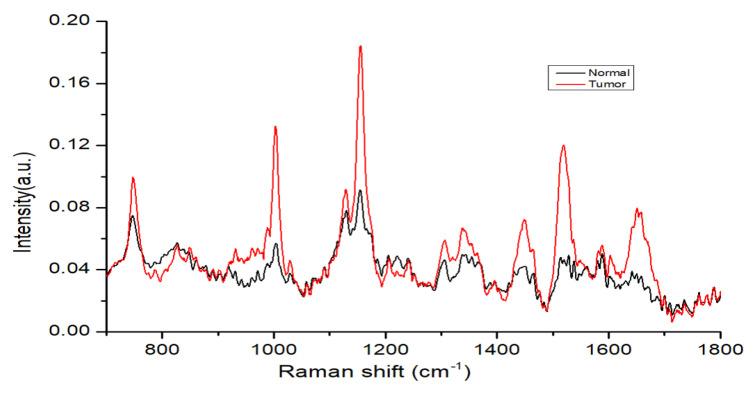
Mean spectra of oral normal and tumor cryopreserved tissues.

Figure 4 shows normalized mean spectra of normal and tumor tissues of the oral mucosa. The fingerprint region from 700 to 1800 cm${}^{-1}$ in biological tissues is rich in proteins, nucleic acids, amino acid, carbohydrates and lipids. According to the existing literature, peaks occurring in normal tissues are lipid-dominated and malignant tissue peaks are protein-dominated [15,16,20,31,32]. In our study, we observed that tumor tissues have higher intensity peaks at 1004, 1156, 1339, 1450, 1523, 1656 cm${}^{-1}$ compared to normal tissues. A sharp and intensive peak at 1004 cm${}^{-1}$ is attributed to the symmetric ring breathing mode of phenylalanine that can be observed in protein-enriched tumor tissue samples, while such a peak in normal tissues is due to hydroxyapatite/phosphate or protein [15,33]. A small peak at 1123 cm${}^{-1}$ in normal tissues is attributed to the C-C skeletal stretch in lipids, and in tumor tissues it is attributed to the C-N stretching mode of protein. A sharp and intense peak at 1155∼56 cm${}^{-1}$ is attributed the protein signal in tumor tissues [34], while it can be attributed to C-C in lipid/proteins in normal tissues. A peak at 1449∼50 cm${}^{-1}$ in tumor tissues indicates protein and is associated with CH${}_{2}$ bending [31,32]; the peak in normal tissues is attributable to CH${}_{2}$ deformation of lipids/collagen. Smaller and wider peaks at 1339 cm${}^{-1}$ in tumor tissues are associated with the adenine feature of nucleic acid/protein [35]. A sharp and more intense peak at 1518∼1524 cm${}^{-1}$ is due to the presence of beta-carotene/nucleic acid and was seen in both type of tissues (normal and tumor) [36]. A broad peak at 1650∼1655 cm${}^{-1}$ is characteristic of proteins in the alpha-helix structure of amide I while in normal tissues, this peak is generated by the C=C bond in lipids or phospholipids [15,32]. The signature of protein, amide I, greater CH${}_{2}$ bending, amide III and amino acid (Tryptophan or phenylalanine) were the main biomolecular difference markers that enabled tumor tissue to be distinguished from normal tissues.

### 3.2. Tissue Fluorescence Feature

A VELscope is a non-invasive light-based detection technique. It utilizes the principle of direct tissue autofluorescence with the blue light excitation wavelength between 400 nm and 460 nm to enhance oral mucosal abnormalities. At these wavelengths, the normal oral mucosa is associated with a pale green fluorescence when viewing through a filter and abnormal tissue is associated with a loss of autofluorescence and appears dark. Oral tissues contain molecules which can have fluoresce (i.e., glow), when excited by light of specific wavelength and each fluorophore has a unique excitation wavelength. Fluorophores are molecules that absorb light at one wavelength and emit light at longer wavelength [37]. The main fluorophores in oral tissues are NADH and FAD, cellular coenzymes, collagen, and elastin within the range of 400–460 nm. Fluorescence visualization (FV) relies on three principles: (1) scattering of light as it interacts with tissue, (2) absorption, and (3) reflection of light from the tissue surface. Which principle is to be existed it depends on biochemical composition of the tissue. Tissue architectural changes in pre-cancerous and cancerous tissues affect their optical properties and can be visualized using autofluorescence and spectroscopic methods [38]. The VELscope exploits the biological characteristic to distinguish healthy tissues from malignant tissue. It has a reasonable sensitivity but is associated with a high number of false-positive results in cases of inflammatory lesions due to the elevated blood flow and concentration of hemoglobin. Hemoglobin in blood also absorbs light and reduces fluorescence in high concentration region. Huang et al. [39] proposed the use of quantitative analysis method to quantify the classification of VELscope images (autofluorescence images) by their intensity and heterogeneity. They used QDA as a method of discriminant analysis classification to differentiate between normal and abnormal (malignant/premalignant) lesions of oral mucosa. They successfully differentiated between abnormal and normal lesions with high specificity and good sensitivity. One major limitation of this approach was evident in differentiating between malignant and premalignant lesions. Therefore, in our previous work, three groups of patients (with normal, premalignant and malignant lesions) were differentiated using multiclass classification method. Information in the image was normalized first and then LDA and QDA were used for classification. In the current work, we used the same normalization method for autofluorescence images.

### 3.3. Autofluorescence Imaging Analysis

**Figure 5:**
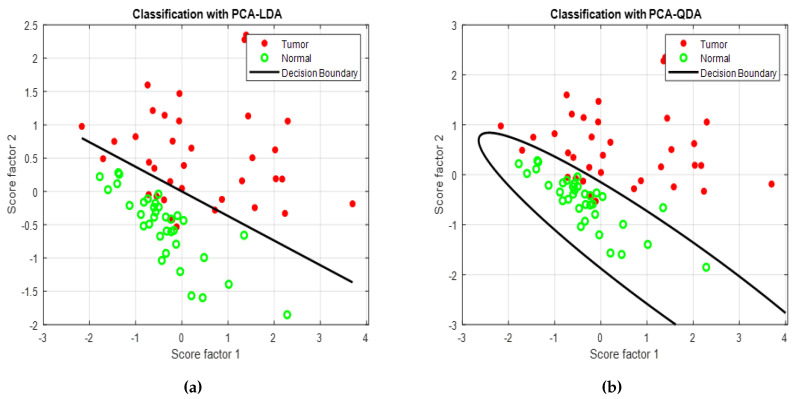
Decision boundary curve for autofluorescence images analysis using (**a**) PCA-LDA and (**b**) PCA- QDA classifier model.

A total of 35 autofluorescence images of malignant samples were collected. The clinician selected two ROIs from each image. The first was from the tumor site and the second was from a site adjacent to the tumor (normal). Normalized intensity and standard deviation were calculated for each ROI. Next, PCA was applied to compress the data. The first two principal components (PC1, PC2) were selected to feed in LDA and QDA classifiers. Figure 5a,b show the scatter plot with linear and quadratic decision boundary curves, where the red and green dots represent tumor and normal tissues, respectively. The PCA–LDA model correctly classified 35/35 and 28/35 tumor and normal sites, respectively. Here, 7 normal cases are incorrectly diagnosed as a tumor by using VELscope with LDA classifier. On the other hand, the PCA–QDA model correctly classified 34/35 and 29/35 tumor and normal samples, respectively, while 6 cases incorrectly diagnosis in the QDA classifier. Both classifier have approximately the same accuracy of about 90%. However, sensitivity and specificity are quite different as shown in Table 2.

### 3.4. Raman Spectroscopic Analysis

**Figure 6:**
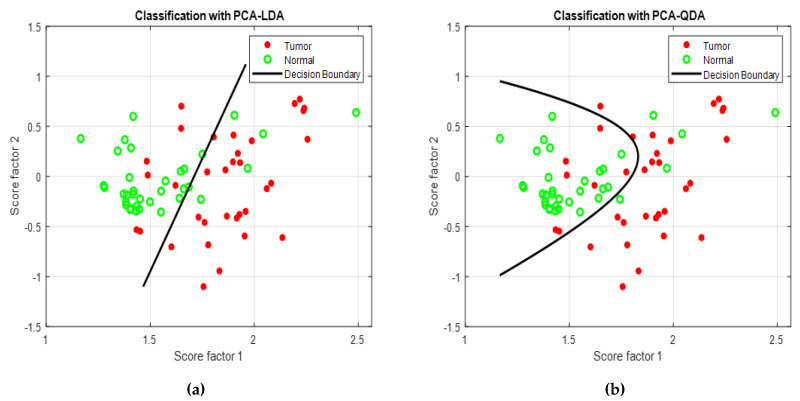
Decision boundary curve for Raman data analysis using (**a**) PCA-LDA and (**b**) PCA-QDA classifier model.

Thirty-five oral cancer patients who had gone through surgical excision were selected. During surgery, 35 tumor tissue samples and 35 tissue samples from adjacent sites were collected. These tissue samples were cryopreserved for Raman analysis. After preprocessing, 35 spectra were collected from both normal and tumor tissues for further analysis. First, PCA was applied on the selected spectrum to examine patterns in the data. Three principle components (PC1, PC2, and PC3) were examined for classification due to giving the maximum variance. PC1 and PC2 were selected to visualize the classification between the samples and feed to LDA and QDA classifiers. Figure 6a,b show the PCA–LDA and PCA–QDA classifier results with decision boundary curve, respectively. These two classifiers generated similar results, as shown in Table 3. Both classifiers have 82.9% accuracy with 85.7% sensitivity and 80% specificity. Here, 7 normal cases are incorrectly diagnosed as a tumor by using Raman data, and 5 tumor cases are incorrectly classified as normal with LDA and QDA classifiers.

### 3.5. Autofluorescence Imaging versus Raman Spectroscopic Analysis

From the autofluorescence images data, the first two principal components (PC1, PC2) were selected, with 100% variance amongst the data are used for input. From Raman data, first two principal components (PC1, PC2) were selected, with 97% variance. In our novel analysis, these four principle components were combined for further analysis and fed in to the LDA and QDA classifiers to improve the accuracy rate. Both models generated the same performance table. However, in our novel analysis, the performance was much better than when using the individual optical techniques (autofluorescence imaging or RS). In combination, they correctly classified 35/35 and 32/35 tumor and normal patients, respectively, and generated higher accuracy 97.14% with maximum sensitivity 100% and better specificity 94.3% as shown in Table 4. Here, only 2 normal cases are incorrectly diagnosed as a tumor by combining autofluorescence imaging with Raman spectroscopic analysis, and no tumor case incorrectly diagnosis as normal. Here, the 100% sensitivity verify the importance of the combination of these two techniques. Because we are providing a complementary study which can be verified by two different technique. If any case VELscope gives some wrong prediction or suspected results in that case we can also justify that by using Raman spectroscopy.

The performance of a model is estimated by cross-validation, a process using a limited number of data samples. It estimates the effectiveness of models (PCA–LDA and PCA–QDA) with unseen data. The sample data set is randomly partitioned into two disjoint subsets. The first one is a training data set and second one is a validation data set. The classification models were trained using the training data set and the validation data set was used to evaluate the performance of the models [40]. The K-Fold and LOOCV (Leave-one-out-cross-validation) methods were used for cross-validation, as previously described in detail [22]. Table 5 shows estimates of the error rates of the model used for Raman data, VELscope data and combination data. The error rates of both PCA–LDA and PCA–QDA models for the Raman data are 17.10%. However, the error rates of both the PCA–LDA and PCA–QDA models for the VELscope data is only 10%. These results are confirmed by the K-Fold and LOOCV methods, which yield error rates of 17.10% and 14.30% for the Raman data, and 10% to both for the VELscope data. After combination the two techniques, the K-Fold and LOOCV methods yield the error rates of 7% and 9%, respectively.

The objective of this study is to combine the two different tumor diagnosis techniques such that a complementary pathway is designed to efficiently diagnose the tumors. Our claim is reflected in Table 5, where the error rate of Raman data analysis with autofluorescence imaging is substantially reduced.

### 3.6. Discussion

The autofluorescence imaging and spectroscopic techniques are fast and non-invasive methods to screen for oral cancer. There are many new optical diagnostic techniques and instruments used in routine clinical practice, such as ViziLite (Zila Pharmaceuticals, Phoenix, AZ, USA), Identafi (DentalEZ, PA, USA), narrow band imaging (NBI; Olympus Medical Systems Corporation, Tokyo, Japan), and VELscope (LED Medical Diagnostics Inc., Burnaby, BC, Canada) [41]. Identafi uses multispectral fluorescence light in a sequential manner to facilitate intraoral examination. NBI is an endoscopic technique to enhance the visualization of oral mucosal abnormalities and underlying vasculature. In NBI, white light is filtered to produce two narrow bands ( 30 nm) of blue light (415 nm) and green light (540 nm). One recent study designed an autofluorescence detection device using two excitation light sources to detect different fluorescent metabolites (375 nm and 460 nm to capture NADH and FAD fluorophores) [42]. In our current study, we utilized two different commercial optical techniques to gain additional and complementary information simultaneously. This was achieved by combining one excitation light (400–460 nm) and one laser source with a wavelength of 532 nm. Light-based detection using VELscope exhibited 90% accuracy with two of the most diagnostically important endogenous fluorophores, NADH and FAD. Other optical techniques, such as the vibrational spectroscopic technique using RS, exhibited 83% accuracy with variation in the main biomolecular difference markers, such as protein structure, amino acids, and beta-carotene. However, our novel approach improved the accuracy rate up to 97% by exploiting the biological characteristics and the biochemical properties simultaneously. Both optical techniques have the potential to differentiate the two classes (normal and tumor), but the combination of the two yielded better results than each individual technique. From Table 2, we can see that the specificities of the VELscope were 80% and 82.6% using PCA–LDA and PCA–QDA. This improved to 94.3% for the combined method (Table 4). Hence, the true negative rate or specificity increased up to 11.4% compared to autofluorescence imaging alone. Specificity is equal to 1—false positive rate (FPR). This means that approximately 20% of cases are still false positives between VELscope and the histology report of our samples. In Table 2, we can see that seven normal cases were incorrectly diagnosed as a tumor from use of the VELscope with LDA while six were incorrectly diagnosed in the QDA classifier. This FPR can be minimized by up to 5.7% in the combined method as well as through use of RS (up to 14%). Thus, we observed that the diagnostic specificity of the VELscope can be improved by our new method.

In this work, our objective was to conduct a combined study, which meant that a sample had to go through the VELscope and RS. We tried to provide an algorithm or method to differentiate between malignancy and normal mucosa. This is why we did not select early stage oral cancer. Once our method proves workable, we may use it in the future to detect early oral cancer. RS is not intended to use directly on patient’s mucosa. We used RS ex-vivo and try to shorten the time of analysis compared with pathology examinations. In a long run, in-vivo Raman analysis can be used but not at this time. Our technique has substantially reduced the FPR. Still, there is one limitation of our study that have a limited number of cases (35 patients with 70 ROIs). To obtain maximum sensitivity and specificity, we need to analyze a huge data set. An inherited limitation of VELscope is that it cannot be easy to capture images of all interior subsites of oral mucosa, including hard palate and retromolar space. However, RS is not bounded by this limitation.

The main advantage of our study is that we can confirm our results by using two different techniques that are completely independent of each other. That is the reason that they can combine to increase sensitivity and specificity. If they were similar, this study could not be reliable or might produce a duplicate testing. Some cases that are suspicious under VELscope examination (false-positive results) can be analyzed or confirmed under RS. Thus, we can verify our skeptical scanning results of VELscope. In this study, we have focused on the same patient who was tested under both techniques while not the same location. Both techniques were performed at a different time but they were surely within the same tumor. For the Raman testing of the samples, we randomly selected different sites of specimens. We assume that the characteristics within the tumor were homogenous. Randomly sampling is to homogenize the differences between testing. So, the testing sites between VELScope and Raman testing could be different in a tumor. Therefore, the results are reliable and comparable.

In the future, we will combine these two techniques for the in vivo application so that these two testing can be done at the same location and time slot. A fiber-optic probe for the Raman system can be used for the in vivo applications in a clinical setting. We can also introduce these two techniques in one portable system by integrating it into the clinical environment by cloud. The iPod will collect the autofluorescence images using VELscope whereas Raman data will be collected by using a fiber-optic probe of the Raman system. These two testings will be done separately. We store the data from both techniques over the cloud, where a clinician can access it for further diagnosis. So, this system can be easily operated by the clinician for oral cancer detection. In the future, it can also probably be used to detect the boundary for surgical resection. We would also further incorporate some artificial-intelligence-based algorithms with VELscopy and RS for tumor invasions.

## 4. Conclusions

In this study, we developed a novel quantitative method to improve oral cancer detection using autofluorescence images and Raman spectra recorded by a VELscope and RS, respectively. The main focus of this study is to differentiate the malignancy and normal mucosa using VELscope and RS. This approach yielded higher sensitivity and specificity with PCA–LDA and PCA–QDA classifier models. The combination of these two quantitative optical tools can create a complementary effect and improve the differentiation of oral tumors. In the future, we will combine these two techniques for the in vivo application so that it can be used as a clinical point of care oral cancer screening device. This novel system can be easily operated by the clinician and any health worker without any formal healthcare training can do oral cancer screening. Further studies are required to confirm the clinical importance of our novel approach. Future investigations will focus on a maximum number of cases in the oral cavity to enhance the classification rate and the use of other approaches that involve meta-learning, neural networks, and combinatorial fusion analysis for improving the classification rate.

## Figures and Tables

**Table 1:** Patient demographics in 35 patients.

Characteristics	N(%)
**Sex**
Male	31 (89)
Female	4 (11)
**Stage**
T1	3 (9)
T2	11 (31)
T3	10 (29)
T4	11 (31)
**Subsites**
Tongue	8 (23)
Buccal mucosa	18 (51)
Gingiva	8 (23)
Mouth floor	1 (3)

**Table 2:** Confusion and performance tables for autofluorescence images (VELscope) analyzed by PCA–LDA and PCA–QDA.

Data Set	Confusion Table			Performance Parameters		
**PCA–LDA**	Normal	Tumor	Total	Accuracy (%)	Sensitivity (%)	Specificity (%)
Normal	**28**	7	35	90	100	80
Tumor	0	**35**	35			
**PCA–QDA**	Normal	Tumor	Total	Accuracy (%)	Sensitivity (%)	Specificity (%)
Normal	**29**	6	35	90	97.14	82.86
Tumor	1	**34**	35			

**Table 3:** Confusion and performance tables for Raman data analyzed by PCA-LDA and PCA-QDA.

Data Set	Confusion Table			Performance Parameters		
**PCA–LDA**	Normal	Tumor	Total	Accuracy (%)	Sensitivity (%)	Specificity (%)
Normal	**28**	7	35	82.9	80	85.71
Tumor	5	**30**	35			
**PCA–QDA**	Normal	Tumor	Total	Accuracy (%)	Sensitivity (%)	Specificity (%)
Normal	**28**	7	35	82.9	80	85.71
Tumor	5	**30**	35			

**Table 4:** Confusion and Performance table of combined study.

Data Set	Confusion Table			Performance Parameters		
**PCA–LDA**	Normal	Tumor	Total	Accuracy (%)	Sensitivity (%)	Specificity (%)
Normal	**33**	2	35	97.14	100	94.3
Tumor	0	**35**	35			
**PCA–QDA**	Normal	Tumor	Total	Accuracy (%)	Sensitivity (%)	Specificity (%)
Normal	**33**	2	35	97.14	100	94.3
Tumor	0	**35**	35			

**Table 5:** Error Rate of PCA–LDA, PCA–QDA and validation methods for different data set.

Error Rate	PCA–LDA (%)	PCA–QDA (%)	Validation: KFold (%)	Validation: LOOCV (%)
Raman analysis	17.10	17.10	17.10	14.30
VELscope analysis	10	10	10	10
Raman+VELscope analysis	3	3	7	9
